# An Analysis of Longan Honey from Taiwan and Thailand Using Flow Cytometry and Physicochemical Analysis

**DOI:** 10.3390/foods13233772

**Published:** 2024-11-25

**Authors:** Lekhnath Kafle, Tandzisile Zine Mabuza

**Affiliations:** Department of Tropical Agriculture and International Cooperation, National Pingtung University of Science and Technology, Pingtung 91201, Taiwan; zine.mabuza@worldveg.org

**Keywords:** longan honey, pollen, forward scatter, side scatter, Y610-A, NUV450, moisture content, honey color, pH, ash content, viscosity

## Abstract

The increase in honey fraud in the global market has highlighted the importance of pollen analysis in determining or confirming the botanical and geographical origins of honey. Numerous studies are currently underway to develop efficient and rapid methods for the determination of the quality, botanical, and geographical origin of honey. Typically, the physicochemical analysis of honey is used to evaluate its quality and geographical source. In this study, flow cytometry, a technique extensively employed in immunology and hematology, was first applied to analyze and characterize pollen from longan honeys from Taiwan and Thailand. The flow cytometry was employed for forward scatter (FSC), side scatter (SSC), Y610-A, and NUV450 to analyze longan honey samples from Taiwan and Thailand. Taiwan’s longan honeys were rich in pollens; however, based upon the FSC and SSC analyses, the pollens from Thai longan honeys were larger and more granular. The Y610/20 emission area was greatest in Thai pollens. The NUV450 measured in the near UV laser was also greater in Thai pollen. Additionally, honey samples were also analysed for physiochemical properties including moisture content, pH, ash content, viscosity, and hydroxymethylfurfural (HMF) for physiochemical properties of longan honey samples from both countries. The moisture content of honey from Taiwan varied between 20.90% and 23.40%, whereas honey from Thailand ranged from 19.50% to 23.50%. A total of 60% of Taiwan’s longan honey was found to have a dark amber color, and only 20% of Thai longan honey exhibited a dark amber color. Furthermore, the pH range of honey from Taiwan was found to be between 4.00 and 4.16, and the pH of Thai honey ranged from 4.01 to 4.12. The ash content of honey samples from Taiwan ranged from 0.05% to 0.23%, and Thai honey had a range of 0.01% to 0.9%. All samples were negative for the Fiehe’s test, indicating the absence of HMF. This analysis lays the groundwork for rapid identification the origins of the honey, applying flow cytometry in conjunction with physicochemical analysis to assess its quality.

## 1. Introduction

Longan honey is one of the most famous and commonly produced monofloral honeys in eastern Asia. The color of longan honey is medium to dark amber. It predominantly serves as a natural sweetener across numerous food products, distinguished by its highly concentrated aqueous solution of inverted sugars, in addition to an assortment of nutrients including saccharides, amino acids, phenolics, flavonoids, vitamins, and minerals [1]. When longan honey was compared with sunflower and wildflower honeys, it was found to contain the highest amount of proline [2]. This may coincide with the strongest smell of longan honey.

According to reports, Taiwan imports most honey from Thailand, and recently the import exceeded the total honey produced locally [3,4]. The longan honey is the most popular honey among Taiwanese consumers and famous for its amber color and aromatic aroma [5]. Due to its high demand and subsequent high prices, concerns regarding the authenticity and adulteration of honey, particularly when it is mixed with cheaper imported longan honey or mislabeled as such, have emerged as significant issues in the market of Taiwan. In such a situation, identifying the source of origin, especially the imported longan honey, is of utmost importance.

In most cases, determining the geographical origin of honey may be complicated [6]. It can be successfully achieved through the identification of predominant and under-represented pollen types. Through the distinction of the unique composition of predominant and secondary or minor pollen grains, the honeys from the same botanical sources can be traced back to their geographical origin. According to Jamil Noor et al. [7], pollen spectra of honey are characteristics of the vegetational composition of specific floras; hence, melissopalynology can also be used to determine the geographical origin of the honey.

Honey quality assessment is done by physiochemical analysis, and the botanical origin of honey is determined by pollens analysis from honey. However, traditional methods of pollen identification, such as microscopy, can be time-consuming and complicated [8,9]. Therefore, scholars are searching for quick and simple methods to analyze the honey to determine its special characters. Flow cytometry has emerged as a promising alternative, offering rapid and objective analysis of pollen grains based on their physical and chemical properties.

The flow cytometry technique is commonly used in the fields of immunology, molecular biology, haematology, and oncology [10]. Flow cytometry utilizes laser-based technology to measure the physical and chemical properties of particles suspended in a fluid stream. Flow cytometry is a powerful technique increasingly applied in pollen analysis, allowing for rapid and precise quantification and characterization of pollen grains [11]. In pollen analysis, flow cytometry enables the differentiation of pollen types based on size, granularity, and fluorescence, making it particularly suitable for pollen identification [12,13,14,15]. The ability to analyze large sample sizes quickly enhances the statistical robustness of ecological studies, making flow cytometry a valuable tool in palynology and related fields [16,17]. Recent advancements have improved the throughput and resolution of flow cytometry, which can distinguish between different pollen types based on their unique morphological and physiological traits [9,18].

In recent years, there has been a significant increase in research employing flow cytometry for the analysis of bee pollen, particularly for applications such as pollen counting, identifying their botanical origins, assessing pollen fitness, and conducting other molecular studies [19,20,21,22,23]. However, to date, no study has specifically focused on extracting pollen from honey and analyzing these samples to identify any differences between honeys derived from different geographical origins but from the same botanical source. Consequently, in this study, we utilized flow cytometry to analyze longan honeys for their special characteristics from Taiwan and Thailand. The goal of this study was to determine whether this method could reveal any variations in pollen characteristics between honeys from these two countries. Given that Taiwan imports the majority of its longan honey from Thailand, it is essential to develop a simple and efficient method for distinguishing between these honeys.

## 2. Materials and Methods

### 2.1. Equipment and Reagents

The equipment and reagents utilized in this study are outlined in Table 1.

### 2.2. Honey Samples

Longan honey samples were obtained from local beekeepers in Taiwan from Tainan, Taichung, Nantou, Chiayi, and Yunlin Counties. Among those areas, Tainan has the highest numbers of longan trees in Taiwan [24]. Thailand longan samples were also obtained from local beekeepers in the northern provinces (Chiang Mai, Chiang Rai, Lampang, Lamphun, and Nan). Among those areas, Chiang Mai has the highest numbers of longan trees in Thailand [25]. The longan honey harvesting season of the northern provinces of Thailand typically occurs between February and March of each year. Similarly, in Taiwan, longan honey is harvested from March to April of each year. From each location in both countries, we collected one kilogram of honey from five beekeepers each. A total of five kilograms of honey, collected from a single location, were thoroughly mixed to prepare a representative sample. One kilogram was then taken as a sample for further studies. All honey samples were produced in February to April 2020. The commercial and artificial honeys were bought from the local market.

### 2.3. Physiochemical Properties

#### 2.3.1. Moisture Content

Moisture content of honey samples was determined following a modified method of Bicudo de Almeida–Muradian, et al. [26] using a honey refractometer (Tiaoyeer Honey Refractometer with ATC, B07GTDY9V2, Mettler Toledo, Columbus, OH, USA). The matted prism surface and illuminator flap were both cleaned prior to usage using a damp cloth, then allowed to dry. Two drops of each honey sample were carefully placed on the clean prism surface, then covered with the cover board. The readings were taken after the apparatus had equilibrated by viewing through the eyepiece. After each measurement, the prism surface and flap were wiped clean with a damp cloth and allowed to dry. The moisture content was observed when the temperature of the homogenized samples was 20 °C. All honey samples were prepared in four replicates.

#### 2.3.2. pH

The potential hydrogen in the honey samples was measured using the pH meter (Hach sensION, PH3 Basic pH Benchtop meter, Hach Company, Loveland, CO, USA). Each 10 mL prepared sample was placed under the electrode stand, then the pH electrode was slowly lowered until it was dipped into the sample. The electrode was wiped with KIMTECH delicate task wipes (Kimberly Clark Taiwan, Taipei, Taiwan) and thoroughly rinsed with distilled water between samples to avoid contamination. Readings were recorded after stable conditions, and the pH was expressed in two decimal places. All honey samples were prepared in triplicates.

#### 2.3.3. Ash Content

To determine the mineral content of honey samples, the modified method of AOAC [27] was used. The porcelain crucibles with lids were washed and rinsed with distilled water and placed in the oven (Hipoint Precision Oven OV-60, Jih Her Tyan Scientific Company Ltd., Kaohsiung, Taiwan) at 550 °C overnight. The desiccator was used to cool the porcelain crucibles for 30 min. Crucibles and lids were weighed, and 5 g of honey was weighed into the crucibles. Heating plates (Corning PC-420D stirring hot plates, P/N 6795-420D, American Laboratory Trading, New London, USA) were used to heat the honey samples to remove moisture. The solidified samples were then heated overnight in the furnace (5.8 L C1 Atmosphere Controlled Ashing furnace, Thermo Scientific, Dreieich, Germany) at 550 °C. Heating of the furnace was conducted in two stages: the first stage of 8 °C/minute up to 98 °C and the second of 6 °C/minute from 98 to 500 °C. Samples were cooled again in a desiccator for 30 min before being weighed. Readings were taken only when the stability detector had disappeared. All honey samples were prepared in triplicates. The Equation (1) below was used to calculate the ash percentage;

Ash (%) = (Wt. of ash/Wt. of sample) × 100(1)

#### 2.3.4. Honey Viscosity

A modified method of Miwa et al. [28] was followed to measure honey viscosity. All measurements were taken at room temperature of 26 °C. Calibration of the viscometer (Sine-wave Vibro Viscometer SV-10, A & D Company Limited, Tokyo, Japan) with measurement limits of 0.3 to 10,000 mPa·s was done with distilled water prior to the experiment. For each measurement, 45 mL of honey in the standard sample cup was placed on the sample platform, then the sensor plate was lowered down until it reached the marked point in the plates. Readings of the viscosity and temperature were taken once the system had stabilized. The experiment was performed in triplicate.

#### 2.3.5. Color Intensity

The color intensity of honey samples was measured following modified methods from previous reports [26,29] according to the Pfund classifier. One 10 mm light path cuvette half-filled with water was used to calibrate the spectrophotometer (DU 730 UV/Vis Spectrophotometer, Beckman’s Coullter, Indianapolis, IN, USA). The wavelength used in this experiment was 560 nm. The wavelength usually used in honey color analysis is ranged from 380 to 740 nm, and many studies used 560 nm [26,30,31,32,33] Therefore, we decided to use the 560 nm wavelength. The absorbance was measured by placing the three-quarters honey-filled cuvette one at a time. The experiment was conducted in four replications. The following Equation (2) was used to convert the absorbance values to the Pfund scale [34];

Pfund = −38.7 + 371.39 × Absorbance (Abs)(2)

According to the approved color standards of the USDA [35], honey with less than 8 mm Pfund values is classified as “water white”, under “extra white” color are those between 9 and 17 mm. Honey of values between 18 and 34 mm is considered “white”. Extra-light amber honey has Pfund values between 35 and 50 mm, while from 51 to 85 mm honey is said to be “light amber”. Amber-colored honey is between 86- and 114-mm honey, and above 114 mm, the honey is classified as dark amber.

#### 2.3.6. Fiehe’s Test

The high levels of hydroxymethylfurfural (HMF) indicate that the honey has been exposed to extensive heat or has been stored for a long period of time. The absence of HMF means that the honey is fresh [36,37]. The presence of HMF in honey samples was determined by Fiehe’s test following a modified method of Bogdanov et al. [38]. The test involved the reaction of HMF with resorcinol to form a cherry red color, which was an indication of the presence of invert sugar, but where HMF was absent, there was no change observed. The steps involved measuring 4 mL of honey and mixing it with 6 mL of ethyl ether (74.12%). The solution was mixed well using a pestle. The supernatant solution was then transferred to a petri dish and allowed to dry in open air. Crystals of resorcinol (1 g) were added to 5 mL of hydrochloric acid (38%) in a test tube, then mixed well to dissolve the powder. A few drops of the resorcinol mixture were then added to the dried supernatant, and color was observed. All samples were prepared in triplicate.

### 2.4. Flow Cytometric Analysis

#### 2.4.1. Pollen Extraction

Pollen was extracted from honey following a modified method of Louveaux et al. [39]. Honey samples were weighed at 15 g each in a 50-mL centrifuge tube. Distilled water was heated in a water bath (Water bath—10 L DSB—500, DIGISYSTEM Laboratory Instruments Inc., Taipei, Taiwan) to 40 °C. The warm water was added to the honey and then mixed with a vortex mixer (Digisystem vortex mixer VM—2000, DIGISYSTEM Laboratory Instruments Inc., Taipei, Taiwan) for 15 s. This was then centrifuged (Thermo Fisher Scientific Megafuge 16R Centrifuge series, Dreieich, Germany) at 1000 r/min for 25 min. The supernatant was decanted off and 15 mL of distilled water added, then the mixer was again centrifuged for 15 min at 1000 r/min. After the clear sediment was obtained, the supernatant was drawn off again to reach 10 mL, then placed in a cooler with ice for further analysis in flow cytometry.

#### 2.4.2. Flow Cytometry

A 200 μL sample was transferred using a pipette into a 5 mL falcon tube, then placed at the opening of the flow cytometric analyzer (CytoFLEX SN2-V4-BY-Y4, Beckman’s Coullter, Indianapolis, IN, USA). The first step was setting up the acquisition and channels. Label names were created under the selected channels. The compensation was set under the compensation matrix tab. Parameters to be analyzed were selected in the electronic system, including forward scatter (FSC), side scatter (SCC), chlorophyll, and DAPI (4′,6-diamidino-2-phenylindole) and also the time (2 min) set for analysis. Gating was performed to discriminate debris and doublets [40]. Samples were mixed with a vortex mixer for 5 s before cytometric analysis. All samples were analyzed for four replications.

### 2.5. Statistical Analysis

Experimental values were expressed as mean ± standard error (SE). Analysis of Variance (ANOVA) and the Tukey HSD test were employed to compare the means obtained in this study, with a significance level set at α = 0.05 (IBM SPSS Statistics version 22.0 (Armonk, New York, NY, USA).

## 3. Results

### 3.1. Physiochemical Properties

#### 3.1.1. Moisture Content

When using a honey refractometer to measure the moisture content of honey samples, we found that the longan honey samples from Lamphun had the highest moisture content among all the samples analyzed, surpassing those from Taiwan and Thailand. However, the moisture content was not significantly higher than that of the Taichung honey sample. Moreover, the moisture content of the Nan honey sample was significantly lower than that of all the honey samples analyzed (F = 1821.50, DF = 10, *p* < 0.01). All honey samples, except for the Nan honey sample, contained a moisture content exceeding 20%, surpassing the national standard set by Taiwan (CNS 1305) [4] (Table 2).

#### 3.1.2. pH

The pH of honey samples was measured using a pH meter. It was observed that the honey sample from Taichung had a significantly higher pH compared to those from Tainan, Yunlin, and Nan. However, it did not significantly differ from those from Nantou, Lam-phun, Lampang, Chiang Rai, and Chiang Mai (F = 4.4, DF = 10, *p* < 0.01). All the honey samples analyzed fell within the acceptable pH range of 3.5 to 5.5 (Table 2).

#### 3.1.3. Ash Content

The ash content of honey was analyzed to determine its mineral composition. The results found that honey from Taichung had a significantly higher ash content compared to honey from Lamphun, Nantou, Lampang, Yunlin, Chiang Mai, and Nan. However, there was no significant difference in ash content among honey samples from Tainan and Chiayi (F = 92.83, DF = 10, *p* < 0.01) (Table 2). Furthermore, the ash content of honey from the Nan sample was significantly lower than all the other samples analyzed. However, it did not differ significantly from the honey samples from Nantou, Yunlin, Lamphun, Lampang, and Chiang Mai (F = 92.83, DF = 10, *p* < 0.01) (Table 2). Sixty and eighty percent of the honey samples from Taiwan and Thailand, respectively, exceeded the minimum allowed mineral content by Taiwanese standards, which is set at less than 0.1%.

#### 3.1.4. Honey Viscosity

The viscosity of honey was measured using a viscometer, and the results revealed that the viscosity of the Nan honey sample was significantly higher than that of all other samples analyzed. Similarly, the viscosity of honey from Chiayi was significantly higher than all samples except for those from Yunlin and Nan. In comparison, the viscosity of honey from Lampang was significantly lower than all the samples analyzed (F = 6665.70, DF = 10, *p* < 0.01). (Table 2). All the honey samples from Taiwan and Thailand fell within the acceptable viscosity range, which was between 2.54 and 23.4 Pa·S.

#### 3.1.5. Color Intensity

The color intensity of all honey samples was analyzed using the Pfund scale, revealing that honey from Taichung exhibited a significantly higher Pfund value compared to all other samples, with the exception of the Yunlin sample. Similarly, there was no significant difference in Pfund value among honey samples from Chiayi, Chiang Mai, Lamphun, and Nan (F = 392.65, DF = 10, *p* < 0.01) (Table 2). We observed that 60% of Taiwanese longan honey was characterized by a dark amber, with the remaining 40% showing either a light amber or amber color. On the other hand, only 20% of Thai longan honey was found to have a dark amber color, with the remaining 80% displaying either a light amber or amber color (Table 3).

#### 3.1.6. Fiehe’s Test

The freshness and presence of adulterants, such as invert sugars, in honey samples were determined using Fiehe’s test. This chemical test is designed to detect the presence of commercial invert sugar or hydroxymethylfurfural (HMF) in honey. Fiehe’s test can determine if honey has been adulterated with commercial or inverted sugars or if it has been aged or overheated [37]. The presence of HMF indicates that honey has been diluted with syrups or artificial honey. According to the report [41], high levels of HMF suggest a lack of freshness and indicate that honey has been repeatedly heated. No red color was observed in any of the honey samples, indicating that all samples analyzed in this study fell within an acceptable range of HMF (Table 3).

### 3.2. Flow Cytometric Analysis

#### 3.2.1. Pollen Count

Most Taiwanese honey samples had a higher average number of pollens compared to Thai samples. Pollen from the Taichung sample had a significantly higher number of pollens among all the longan honey samples analyzed. Similarly, Nantou sample had significantly higher pollen particles than all of Thai honey samples. The pollen count of the longan honey sample from Chiayi was significantly lower than the pollen count among all the honey samples analyzed, however, it was not significantly different from the Chiang Rai and Nan samples (F = 170.29, DF = 9, *p*< 0.01) (Table 4).

#### 3.2.2. Y610/20 Fluorescence Emission

The green-yellow laser efficiently excited and detected the chlorophyll protein at 561 nm emitting fluorescence in the Y610/20 channel of Taiwanese and Thai honey samples. The comparison of Y610/20 fluorescence emitted by pollen from Taiwan and Thai longan honey samples showed slight differences. The Nan honey sample had pollen with the significantly highest Y610/20 fluorescence of all the samples analyzed. The emitted fluorescence of pollen from the Yunlin honey sample was not significantly different from the honey from Chiang Mai; however, it was significantly higher than the rest of the samples. Furthermore, the Tainan honey sample had the lowest Y610/20 fluorescence among all the samples analyzed; however, that was not statistically different from the Nantou, Chiayi, Chiang Rai, and Lamphun honey samples (F = 57.32, DF = 9, *p*< 0.01) (Table 4).

#### 3.2.3. NUV450 Fluorescence Emission

Pollen and other biological structures absorb and emit light, which is then used to distinguish their features. The near ultraviolet (NUV) laser-excited pollen labeled with DAPI is a DNA-binding and UV-stimulated probe used in the determination of nuclei quantities and assessment of gross cell morphology [13,42]. Pollens were excited at 375 nm and emitted 450/50 fluorescence.

There were slight differences observed in the NUV450 pollen fluorescence from Taiwanese and Thai longan honey samples. Pollen from the Nan honey sample significantly emitted the highest fluorescence than all other samples analyzed. Likewise, the NUV450 fluorescence of the Lampang honey sample was significantly higher than all samples except for the Tainan, Chiang Rai, and Yunlin honey samples that were not statistically different. Furthermore, NUV450 emission for pollen from Nantou honey sample was significantly lower than Nan, Lamphun, and Tainan samples but was not significantly different from the rest of the samples analyzed (F = 110.54, DF = 9, *p*< 0.01) (Table 4).

#### 3.2.4. Forward Scatter

The forward scatter (FSC) gives information of an individual pollen grain as it passes the interrogation point at a certain angle of light, and this measures its size [43,44,45,46]. There were significant differences in the FSC of pollen extracted from Taiwanese and Thai longan honey samples, indicating a difference in the size of pollen in the different samples. The Nan honey sample’s pollen had a significantly higher FSC than all analyzed samples. The FSC for Yunlin, Chiang Mai or Taichung, Lamphun, and Chiang Rai or Chiayi, and Tainan honey samples were not significantly different. The Nantou sample had the significantly lowest pollen FSC than all other samples analyzed (F = 163.75, DF = 9, *p*< 0.01) (Table 5).

#### 3.2.5. Side Scatter

The side scatter signal (SSC), which measures cell granularity, is usually plotted in correspondence with FSC [44,45,46]. The granularity of pollen from Taiwanese and Thai longan honey samples, as indicated by the SSC, was mostly not significantly different across samples. The SSC fluorescence for pollen from the Nan honey sample again was significantly higher compared to the rest of the samples. Pollen SSC of Lampang and Yunlin or Chiang Mai, Lamphun, and Tainan or Chiang Rai, Taichung, Nantou, and Chiayi honey samples were not significantly different (F = 41.05, DF = 9, *p*< 0.01) (Table 5).

#### 3.2.6. Pollen Distribution of Taiwanese and Thai Longan Honey

**Figure 1:**
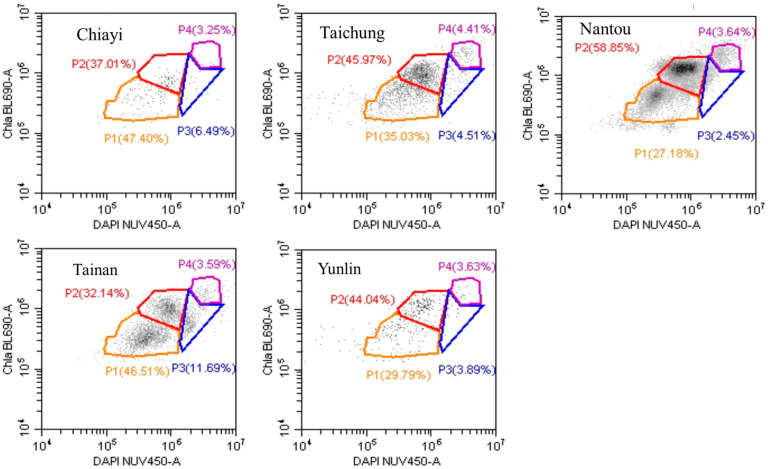
Scatter plots of pollen from Taiwanese longan honey samples. The fluorescent stain 4′,6-diamidino-2-phenylindole (DAPI) and chlorophyll protein (Chlorophyll a BL690-A) were used to create subpopulations. P1, P2, P3, and P4 indicated four randomly selected subpopulations of pollen. Chla BL690-A = Chlorophyll a BL690-A. DAPI NUV450-A = DAPI NUV450-A Nucleic Acid Stain.

Figure 1 illustrates the distribution and proportions of selected subpopulations of pollen of longan honey from Taiwanese and Thailand. The fluorescent stain 4′,6-diamidino-2-phenylindole (DAPI) and chlorophyll protein (Chlorophyll a BL690-A) were used to create subpopulations. Four regions (P1, P2, P3, and P4) were selected in the plots to represent subpopulations, which are present in different proportions in longan honey samples. The Chiayi sample’s pollen was mostly classified under P1, and the second highest population was P2. Only a few pollens in the Chiayi sample belonged to P4. Tainan also had the highest percentage of pollen under P1, which was followed by P2, P3, and P4. Subpopulation 2 was highest in Nantou, Taichung, and Yunlin samples, while P1 had the second highest percentage of pollen. In addition, the lowest proportion of pollen in Taichung and Yunlin samples belonged to P4. In the Nantou sample however, the least number of pollens was for P3 (Figure 1).

**Figure 2:**
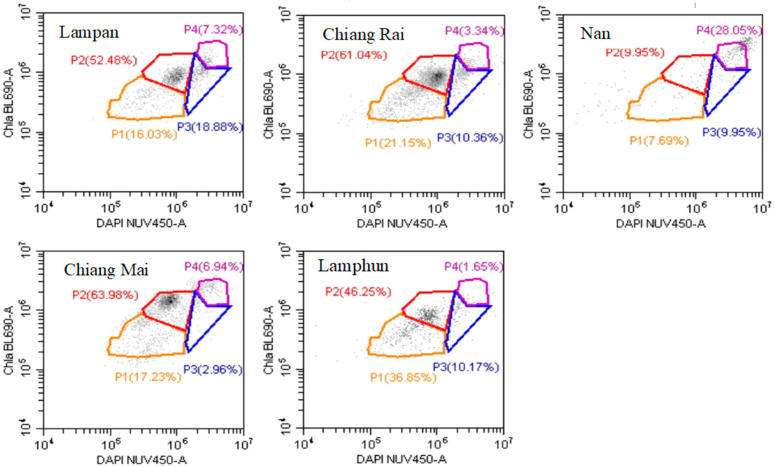
Pollen subpopulations of Thai longan honey samples analyzed by scatter plots. The fluorescent stain 4′,6-diamidino-2-phenylindole (DAPI) and chlorophyll protein (Chlorophyll a BL690-A) were used to create subpopulations. P1, P2, P3, and P4 indicated four randomly selected subpopulations of pollen. Chla BL690-A = Chlorophyll a BL690-A. DAPI NUV450-A = DAPI NUV450-A Nucleic Acid Stain.

The highest pollen subpopulation in the Lampang sample was P2, followed by P3, P1, and P4. Similarly, the sample from Chiang Rai had the highest percentage of pollen classified under P2, while P4 had the lowest pollen. Subpopulation 4 was the highest in the Nan sample, while P2 and P3 were in equal proportion. The Chiang Mai sample had the most of its pollen classified as P2, whereas the least number of pollens were in P3. Furthermore, pollen from the Lamphun sample was highest in P2, followed by P1, P3, and P4. Subpopulation 2 had the highest number of pollens in all Thai honey samples except in the Nan sample. The P4 had lower pollen in all samples except in the samples from Nan (Figure 2).

Table 6 shows the post-hoc analysis of P1, P2, P3, and P4 subpopulations for Taiwanese and Thai pollen extracted from different longan honeys. There was a significant difference (*p* < 0.05) when Taiwan’s P2 was compared with any of the other subpopulations. There was no significant difference in the proportions of subpopulations 1, 2, 3, and 4 when Thai samples were compared with each other. Taiwan’s P2 was significantly higher compared with Thai P1, P2, P3, and P4 (Table 6).

Subpopulation 2 of Taiwan samples was significantly higher than P1, P3, and P4 of Taiwan’s samples. There was no significant difference between the subpopulations P1, P3, and P4 of both Taiwanese and Thai samples; however, P2 was significantly different from all subpopulations. This means that the selected subpopulations were present in almost equal proportions in Taiwanese and Thai longan honey samples except for P2 (Table 6).

#### 3.2.7. Pollen Count, FSC, and SSC of Artificial and Commercial Longan Honeys

Upon conducting a thorough analysis of the Taiwanese and Thai longan honeys through flow cytometry and phisiochemical analysis, we also undertook the examination of pollen count, FSC, and SSC analysis of the longan honeys available in the local market of Taiwan. These parameters were subsequently compared with those of the artificial (artificial or fake) longan honey. The pollen numbers of Taiwan longan honey were significantly different than the Thai longan honey samples. Taiwan longan honey samples had approximately 11 folds higher pollen numbers than the Thai longan honey samples. The number of pollens of Taiwanese and Thai longan honey samples was significantly higher than the artificial honey (F = 201.356, DF = 2, *p*< 0.01) (Table 7).

Similarly, FSC, and SSC values of Taiwanese longan honey were significantly higher than the Thai longan honey samples, and the FSC and SSC values of both honey samples were significantly higher than the artificial honey. The FSC and SSC values of Taiwan longan honey were 1.1 and 1.3 folds higher than the Thai longan honey samples (FSC: F = 534.61, DF = 2, *p*< 0.01; SSC: F = 377.20, DF = 2, *p*< 0.01) (Table 7).

**Figure 3:**
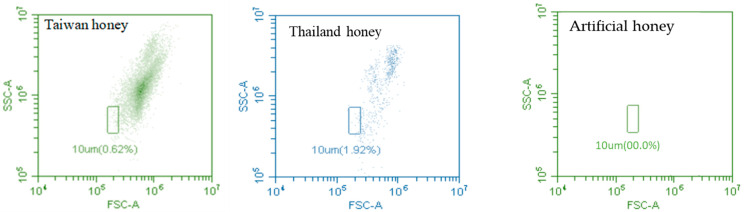
Dot plot for showing pollen distribution of Taiwan, Thai longan honeys, and artificial honey. The pollen is as measured in the forward (FSC) and side scatter (SSC); the area marked 10 μm excludes particles or debris.

Figure 3 shows the distribution of pollen as detected by the FSC and SSC of the commercial Taiwanese and Thai longan honeys. No pollen was present in the artificial honey. Debris (<10 μm) were present in a very low percentage, which indicates that pollen was not damaged during extraction.

## 4. Discussion

Physicochemical parameters of honey, such as pH, viscosity, moisture content, ash content, color intensity, and HMF level, have been studied extensively to characterize honeys from different regions. Monitoring those properties are very important because of the reactions that continue to occur even in storage; the properties of honey change, indicating a reduction in its quality [47,48].

According to Taiwan’s national standard (CNS 1305) for honey, the moisture content for longan honey should be no more than 20% [4,49]. Most of the samples from Taiwan or Thailand had a moisture content above 20%, and only the Nan (Thailand) sample has a moisture content within the acceptable level. The difference in the moisture content between the samples may be due to the differences in the moisture content of the natural nectar source and different handling processes. This means that even though the samples are monofloral and from the same location, the parameters may still vary.

Generally, all honeys are acidic, with pH ranging between 2.6 and 6.3. Fermentation and honey ripening also may influence the amount of acids in honey. Honey that is diluted with syrups has low acidity, whereas honey adulterated with invert sugar usually has high acidity [50]. The proportion and composition of organic acids naturally present in honey vary depending on the floral sources and the bee species. The honey pH variations may also be due to the harvested season, geographical origin, period of storage, and mineral content [51,52]. All honey samples from Taiwan or Thailand had a pH within the acceptable range.

The 60 and 20% of the longan honey samples from Taiwan and Thailand, respectively, had their mineral content higher than the minimum level allowed by both the Taiwanese and Thai standards (<0.1%) [4,49]. The mineral composition of honey is markedly influenced by the variety of floral sources and the geographical location of the honey production area [53,54].

According to Bogdanov et al. [55], color determination is an important classification criterion for monofloral honeys. Darker-colored honeys usually have relatively higher conductivity than bright-colored honeys. According to Srisayam and Chantawannakul [56], Thai longan honey was only classified as amber and brown amber. These results differ with the present study, as two samples from Thailand were classified as dark amber. According to Sakdatorn [57], the optical properties of honey can be influenced by substances or particles present in the honey hence, reducing the particle sizes, which can increase the lighter and redder values. González–Miret et al. [58] studied the correlation between the mineral content and color in several honeys by using multivariate statistical methods, including multiple linear regression. They reported that there was a strong correlation between the color of darker honeys and the abundance of calcium and trace elements such as Fe, S, Pb, Cd, and As.

Since the values of the Pfund scale are derived from the absorbance, all absorbance values correspond with the Pfund scale values. A low Pfund value means that the honey sample is likely to be classified as light in the color grader; on the other hand, honey with a higher Pfund scale value would be classified as dark [59]. Both the low and high Pfund values were observed in Thai samples.

Most of the honey samples used in this study were dark amber or light amber by the Pfund classification. The highest percentage of analyzed honey samples were observed in dark amber-colored honey, which was 45.5%. Out of five samples that were dark amber, three were from Taiwan and two from Thailand. The darker color of honey is associated with richness in mineral content, especially iron [53,54,58]. A study by Sant’Ana, Ferreira, Lorenzon, Berbara, and Castro [34] showed that there is a correlation between the total flavonoids and color intensity. Samples classified as light amber in color were 27.3%, and the lowest percentage of honey samples was 9.1% for extra light amber. Between the two longan honey samples that were light amber were from Chiang Rai and Chiayi. Only the Nan longan honey sample was classified as extra-light amber. Two honey samples classified as amber in color accounted for 18.2% and were from Tainan and Lamphun. In general, Taiwan’s longan honeys were darker in color compared with longan honey from Thailand. Although the color of longan honey may vary depending on the place of origin, most studies have classified it as amber, Moniruzzaman et al. [60].

The viscosity of honey decreases when temperature or moisture content of honey increases because of the reduction of hydrodynamic forces and molecular friction [61,62]. Honey’s viscosity varies with different floral sources, even when they have similar moisture content. The presence of crystals and colloids, such as proteins, in honey increases viscosity. Honey that has increased fructose concentration turns out to be less viscous [63]. The differences observed in the viscosities of Taiwanese and Thai longan honeys may be due to the factors that influence viscosity, including the presence of colloids, water content, and chemical constitutions. All the longan honey samples analyzed were within the acceptable range, which is between 2.54 and 23.4 Pa·S.

Bee pollens had been analyzed by flow cytometry in many studies [11,12,13,14,15,19,20,21,22,23]. However, pollens were successfully extracted from all longan honey samples analyzed in this study. Pollen are important in tracing the botanical and geographical origin [64]; therefore, pollen should not be filtered from honey [65,66]. Chiayi and Tainan counties are located in south-western Taiwan, and longa honey samples from both areas had fewer pollens. On the contrary, longan honey samples from Taichung, Nantou, and Yunlin located in the central part of Taiwan contained rich pollens. A report [5] stated that Taiwan’s climatic conditions favor the growth of diverse plant fauna, which are rich nectar sources. The main nectar sources—longan, lychee, and citrus—are concentrated in the central and southern parts of Taiwan [4,5]. The rich pollen from Nantou and Taichung samples may be attributed to the abundance of floral sources in those areas.

The Thai honey samples from Chiang Mai, Lampang, and Lamphun were richer in pollen than the other two areas. In Thailand, longan honey production is concentrated in the upper northern provinces, especially the Chiang Mai province, which is a part of the honey zone producing a significant amount of longan honey. The northern provinces of Thailand have both agricultural and non-agricultural areas that provide the nectar sources for honey bees, especially longan, corn, mangoes, and mung bean [67]. Corn is the major pollen source in Thailand, as it is available almost all year round, while the nectar-flow season normally commences late February up to mid-April, which is the same period for longan bloom. According to Chantawannakul [68], *Bidens pilosa* was the major pollen source in the Chiang Mai province, Thailand; however, there are even more plant species, including *Mimosa pudica*, coffee, and tea, contributing to pollen resources. A study [67] conducted in 2019 reported the main possible nectar sources in Thailand’s Northern Province as forest trees and agricultural crops since these two dominate the land use. Sometimes, to make their honey lighter colored and clear, beekeepers preferred to filter the honey. Such practices also cause fewer numbers of pollens in the honey [69].

Among the Taiwanese longan honey, Yunlin honey has relatively greater FSC values. Moreover, the pollens from Thai honey samples had greater FSC values compared to pollens from Taiwanese honey samples, even though generally Taiwanese longan honey samples had more pollens. Among the Thai longan honey samples, Nan, Chiang Mai, and Lampang had greater FSC values. That means the pollen from Thai honey samples was relatively larger than the pollen from Taiwanese samples. In flow cytometry, the measurement of FSC enables the differentiation of cells based on size, as the intensity of FSC is directly proportional to the diameter of the cell [43,44,45,46].

Among the Taiwanese longan honeys, the honey from Yunlin exhibited notably higher SSC values. Similarly, among the samples of Thai longan honey, those from Nan, Chiang Mai, and Lampang were found to possess higher SSC values. Therefore, the majority of pollens from Thailand were more granular than Taiwanese pollen. The SSC value measures the cell granularity [44,45,46].

The green–yellow laser effectively excited and detected the chlorophyll protein at 561 nm, resulting in fluorescence emission in the Y610/20 channel. The Nan honey sample exhibited pollen with the highest Y610/20 fluorescence, surpassing all other samples, including those from Chiang Mai and Lamphun. The samples from Yunlin had pollen with the highest Y610/20 fluorescence among all samples from Taiwan. The mean emission value in the Y610/20 channel did not show a direct correlation with the number of pollen particles; however, there was a correlation with the FSC and SSC values. Studies reported that in which cells exhibited a low fluorescence signal and were smaller in size, and in which the cells displayed a higher fluorescence signal and were larger [70]. That is the reason that the emission value in the Y610/20 channel was directly correlated with the FSC and SSC values.

Even all the honeys were from the same longan; however, different observations were observed for FSC and SSC, NUV450, and Y610/20 fluorescence. Pöhlker and colleagues [71] revealed a remarkable heterogeneity in fluorescence intensity and emission wavelength across pollen grains of the same species. This suggests that the fluorescence micro-architecture of pollen grains is highly complex and likely influenced significantly by maturation and metabolic state. The most characteristic fluorescence originates from cell wall-associated phenolics and carotenoids. The researchers [71] found that cell wall-associated fluorophores dominate the fluorescence signatures of dry pollen. In another study [13], it was concluded that intensity of fluorescence increases with pollen age. Consequently, pollen from Taiwan honey samples exhibited dissimilar features. However, the mean value for the NUV450 followed a very close sequence with the FSC and SSC fluorescent signals.

In our study, we found that the commercial longan honey samples from Taiwan contained approximately 11 times more pollen compared to those from Thailand. If honey is filtered and pasteurized, the number of pollen particles decreases. Many beekeepers prefer to filter their honey to achieve a clearer consistency. This process enables them to eliminate certain impurities and, more importantly, to remove a significant amount of pollen particles from the honey [69,72]. Based on our findings, we hypothesized that Thai longan honey undergoes more filtration processes than Taiwanese longan honey.

Similarly, the FSC value of Taiwanese commercial longan honey was significantly higher than that of Thai commercial longan honey. Based on the FSC results, it can be concluded that the pollen sizes in Thai longan honey were larger than those in Taiwanese commercial longan honey. In flow cytometry, the measurement of FSC allows for the differentiation of cells based on size [43,44,45,46].

On the other hand, the SSC values of Thai commercial longan honey were significantly higher than those of Taiwanese commercial longan honey. Based on the SSC results, it can be concluded that the pollen particles in Thai longan honey were more granular than those in Taiwanese commercial longan honey. This is because the SSC value measures the granularity of the pollen cells [44,45,46].

Flow cytometric methodologies have been employed to distinguish pollen species by analyzing DNA quantity, scatter, and fluorescence characteristics [13,22,73]. Flow cytometry offers superior capture efficiencies compared to manual microscopy, enabling the rapid processing of pollen grains. Moreover, it has been reported that the accurate identification of pollen species is contingent upon the utilization of flow cytometry in conjunction with microscopy techniques [13,19]. A research group [13] has developed an innovative method for identifying and counting pollen using automated multispectral imaging flow cytometry in combination with deep learning. This approach opens up possibilities for further studies, such as determining the minor pollen species in longan honeys from different countries. By integrating imaging flow cytometry with deep learning, it is possible to gain deeper insights into the pollen diversity of both monofloral and multiflora honeys, as well as to identify the botanical and geographical origins of honeys.

## 5. Conclusions

Based on our observations from flow cytometry, we found that Taiwan’s longan honey was richer in pollen than longan honey from Thailand. The FSC and SSC values of Thai honey were higher than Taiwan honey, which means pollens were relatively larger and more granular in Thai honey. The physicochemical properties of longan honey from Taiwan and Thailand varied. All the samples from Taiwan longan honey had a moisture content higher than the limit for local honey regulations; however, only one Thai sample was above the limit. We found that 60% of Taiwan’s longan honey has a dark amber, with the remaining 40% showing either a light amber or amber color. Conversely, only 20% of Thai longan honey was found to have a dark amber color, with the remaining 80% displaying either a light amber or amber color. The pH of all honey samples used in this study was within the acceptable range according to the standards of both Taiwan and Thailand. The 60 and 20% longan honey samples from Taiwan and Thailand, respectively, exceeded the maximum limit of ash contents allowed by the Taiwanese standard. The viscosity was within the acceptable values in both Taiwan and Thai longan honeys. None of the honey samples was positive for the Fiehe’s test, indicating acceptable levels of HMF. While some studies have utilized flow cytometry for bee pollen analysis, to our knowledge, no research has applied this technique to analyze pollen in honey for the purpose of characterizing honey from different origins. In our study, we successfully characterized the longan honey from both Taiwan and Thailand through flow cytometric and physicochemical analysis. However, we were unable to identify the species of all the pollen present in the honey samples. A more detailed study could be planned to analyze honey from a broader area of both countries with a larger number of samples and to develop a quick and simple flow cytometric protocol for authenticating longan honey from Taiwan and Thailand.

## Figures and Tables

**Table 1:** Equipment and reagents used during study.

Name	Model	Manufacturer
Flow cytometry analyser	CytoFLEX SN2-V4-BY-Y4	Beckman’s Coullter, Indianapolis, IN, USA
CytExpert software	CytExpert software, V 2.4	Beckman’s Coullter, Indianapolis, IN, USA
Spectrophotometer	DU 730 UV/Vis	Beckman’s Coullter, Indianapolis, IN, USA
PH3 Basic pH Benchtop meter	Hach sensION	Hach Company, Loveland, CO, USA
Honey refractometer	Tiaoyeer B07GTDY9V2	Mettler Toledo, Columbus, OH, USA
Hot pan	P/N 6795-420D	American Laboratory Trading, New London, CT, USA
Ashing furnace	5.8 L C1	Thermo Fisher Scientific, Dreieich, Germany
Centrifuge	Megafuge 16R	Thermo Fisher Scientific, Dreieich, Germany
Analytical balance	AB 204-S	Mettler Toledo, Giessen, Germany
Hipoint precision oven	OV-60	Jih Her Tyan Scientific Company Ltd., Kaohsiung, Taiwan
Digisystem vortex mixer	VM—2000	Digisystem Laboratory Instruments Inc., New Taipei City, Taiwan
Water bath	10 L DSB—500	Digisystem Laboratory Instruments Inc., New Taipei City, Taiwan
Balance	VIBRA HJ-220E	Shinko Denshi Company, Tokyo, Japan
Vibro viscometer	SV-10	A & D Company Limited, Tokyo, Japan
Hydrochloric acid (38%)	-	Avantor Performance Materials, LLC., Radnor, PA, USA
Resorcinol powder (C_5_H_6_O_2_)	-	Xilong Scientific Company Ltd., Guangzhou, China
Ethyl Ether (74.12%)	-	Juxing Chemical Instrument Company Limited, Kaohsiung, Taiwan

**Table 2:** Moisture content, pH, ash content, and viscosity of Taiwanese and Thai longan honey samples.

Sample Origin	Parameters (M ± SE) *			
	Moisture Content (%)	pH	Ash Content (%)	Viscosity (Pa·s)
Taiwan				
Chiayi	20.90 ± 0.03 ^e^	4.07 ± 0.02 ^ab^	0.15 ± 0.02 ^bcd^	6.97 ± 0.01 ^b^
Nantou	22.00 ± 0.03 ^c^	4.14 ± 0.04 ^ab^	0.08 ± 0.02 ^cde^	4.51 ± 0.02 ^f^
Taichung	23.40 ± 0.00 ^a^	4.16 ± 0.01 ^a^	0.23 ± 0.01 ^b^	3.41 ± 0.01 ^h^
Tainan	22.50 ± 0.03 ^b^	4.00 ± 0.02 ^b^	0.19 ± 0.01 ^bc^	4.31 ± 0.01 ^g^
Yunlin	21.50 ± 0.03 ^d^	4.03 ± 0.04 ^b^	0.05 ± 0.02 ^de^	6.96 ± 0.07 ^b^
Thailand				
Chiang Mai	20.90 ± 0.10 ^e^	4.07± 0.06 ^ab^	0.03 ± 0.00 ^de^	6.42 ± 0.01 ^d^
Chiang Rai	20.50 ± 0.00 ^f^	4.09 ± 0.01 ^ab^	0.90 ± 0.08 ^a^	6.82 ± 0.02 ^c^
Lampang	21.00 ± 0.00 ^e^	4.12 ± 0.05 ^ab^	0.06 ± 0.00 ^de^	5.49 ± 0.01 ^e^
Lamphun	23.50 ± 0.00 ^a^	4.09 ± 0.03 ^ab^	0.08 ± 0.01 ^cde^	2.72 ± 0.01 ^i^
Nan	19.50 ± 0.03 ^g^	4.01 ± 0.01 ^b^	0.01 ± 0.00 ^e^	9.46 ± 0.01 ^a^

* Means within a column followed by the same letter are not significantly different (α = 0.05) (Tukey HSD test). M = Mean, SE = Standard error, n = 44.

**Table 3:** Absorbance, Pfund scale, color, and HMF for Taiwanese and Thai longan honey samples.

Sample Origin	Absorbance (a.u)	Pfund Scale (mm) (M ± SE) *	Color of Honey Based upon Pfund Scale	HMF Detection
Taiwan				
Chiayi	0.25 ± 0.01	81.426 ± 1.46 ^f^	Light amber	Negative
Nantou	0.35 ± 0.01	117.107 ± 0.76 ^d^	Dark amber	Negative
Taichung	0.47 ± 0.01	154.867 ± 2.00 ^b^	Dark amber	Negative
Tainan	0.29 ± 0.01	97.146 ± 1.34 ^e^	Amber	Negative
Yunlin	0.45 ± 0.03	149.627 ± 4.42 ^b^	Dark amber	Negative
Thailand				
Chiang Mai	0.55 ± 0.02	183.146 ± 3.21 ^a^	Dark amber	Negative
Chiang Rai	0.25 ± 0.00	82.258 ± 0.67 ^f^	Light amber	Negative
Lampang	0.39 ± 0.01	128.335 ± 0.76 ^c^	Amber	Negative
Lamphun	0.26 ± 0.01	86.749 ± 1.81 ^f^	Amber	Negative
Nan	0.15 ± 0.01	50.818 ± 0.85 ^g^	Light amber	Negative

* Means within a column followed by the same letter are not significantly different (α = 0.05) (Tukey HSD test). A.U = Absorbance units. Pfund value was calculated from absorbance value with the formula: Pfund = −38.7 + 371.39 × Absorbance (Abs). M = mean, SE = standard error, n = 44. HMF = Hydroxymethylfurfural.

**Table 4:** Pollen count, Y610/20 fluorescence emission, and NUV450 for Taiwanese and Thai longan honey samples.

Sample Origin	Pollen Count (M ± SE) *	Y610/20 (a.u) (M ± SE) ^α^	NUV450 (a.u) (M ± SE) ^β^
Taiwan			
Chiayi	181.00 ± 28.10 ^f^	4.81 × 10^5^ ± 6.66 × 10^3 e^	9.25 × 10^5^ ± 8.50 × 10^3 cd^
Nantou	9853.25 ± 434.22 ^b^	5.47 × 10^5^ ± 4.12 × 10^3 de^	7.93 × 10^5^ ± 1.51 × 10^4 d^
Taichung	12,276.50 ± 368.88 ^a^	6.01 × 10^5^ ± 7.12 × 10^3 cd^	9.32 × 10^5^ ± 5.34 × 10^4 cd^
Tainan	1986.25 ± 148.72 ^de^	4.40 × 10^5^ ± 1.07 × 10^4 e^	1.11 × 10^6^ ± 5.34 × 10^4 bc^
Yunlin	4038.75 ± 115.53 ^c^	7.11 × 10^5^ ± 5.07 × 10^3 bc^	1.03 × 10^6^ ± 3.84 × 10^4 bcd^
Thailand			
Chiang Mai	2313.25 ± 166.72 ^d^	7.78 × 10^5^ ± 4.87 × 10^3 b^	1.30 × 10^6^ ± 3.97 × 10^4 cd^
Chiang Rai	654.50 ± 43.99 ^ef^	4.72 × 10^5^ ± 2.77 × 10^4 e^	8.25 × 10^5^ ± 8.52 × 10^3 bcd^
Lampang	2553.25 ± 752.72 ^cd^	6.34 × 10^5^ ± 1.44 × 10^4 cd^	1.04 × 10^6^ ± 1.20 × 10^4 b^
Lamphun	2230.00 ± 169.39 ^d^	5.50 × 10^5^ ± 1.20 × 10^4 de^	9.10 × 10^5^ ± 1.07 × 10^4 cd^
Nan	594.25 ± 21.23 ^ef^	1.06 × 10^6^ ± 6.84 × 10^4 a^	2.90 × 10^6^ ± 1.68 × 10^4 a^

^α^ Y610/20 is the channel from which the green-yellow laser emits fluorescence for long-wavelength fluorescent proteins. a.u = arbitrary units. ^β^ NUV450 is the channel from which the near UV laser detected and measured fluorescence of 4′,6-diamidino-2-phenylindole (DAPI). * Means within a column followed by the same letter are not significantly different (α = 0.05) (Tukey HSD test). M = mean, SE = standard error, n = 40.

**Table 5:** Forward scattered (FSC) and side scattered (SSC) of Taiwanese and Thai longan honey samples.

Place of Origin	FSC (a.u) (M ± SE) *	SSC (a.u) (M ± SE) *
Taiwan		
Chiayi	4.45 × 10^5^ ± 4.82 × 10^3 ef^	1.12 × 10^6 ±^ 1.27 × 10^5 e^
Nantou	4.20 × 10^5^ ± 2.25 × 10^3 g^	1.01 × 10^6^ ± 1.61 × 10^5 e^
Taichung	4.65 × 10^5^ ± 2.15 × 10^3 d^	1.01 × 10^6^ ± 2.12 × 105 ^e^
Tainan	4.43 × 10^5^ ± 3.14 × 10^3 f^	1.25 × 10^6^ ± 5.98 × 10^4 de^
Yunlin	5.37 × 10^5^ ± 3.18 × 10^3 b^	1.35 × 10^6^ ± 2.04 × 10^5 bc^
Thailand		
Chiang Mai	5.34 × 10^5^ ± 6.16 × 10^3 b^	1.50 × 10^6^ ± 1.25 × 10^4 cd^
Chiang Rai	4.57 × 10^5^ ± 6.40 × 10^3 def^	1.11 × 10^6^ ± 2.66 × 10^4 e^
Lampang	5.12 × 10^5^ ± 3.10 × 10^3 c^	1.31 × 10^6^ ± 2.04 × 10^4 b^
Lamphun	4.64 × 10^5^ ± 4.27 × 10^3 de^	1.24 × 10^6^ ± 3.08 × 10^4 de^
Nan	5.87 × 10^5^ ± 3.63 × 10^3 a^	1.65 × 10^6^ ± 1.77 × 10^4 a^

FSC = forward scattered fluorescence measuring the size of pollen; SSC = side scattered fluorescence indicating pollen granularity. a.u = arbitrary units. * Means within a column followed by the same letter are not significantly different (α = 0.05) (Tukey HSD test). M = mean, SE = standard error, n = 40.

**Table 6:** Post- Hoc for Taiwanese and Thai subpopulations (after four subpopulations were selected in the scatter plots and named P1, P2, P3, and P4).

Country	Subgroup	Thailand				Taiwan			
		P1	P2	P3	P4	P1	P2	P3	P4
Thailand	P1		0.999	1.000	1.000	0.866	0.000	1.000	1.000
	P2	0.999		0.994	0.993	0.996	0.001	0.999	1.000
	P3	1.000	0.994		1.000	0.787	0.000	1.000	1.000
	P4	1.000	0.993	1.000		0.774	0.000	1.000	1.000
Taiwan	P1	0.866	0.996	0.787	0.774		0.027	0.866	0.953
	P2	0.000	0.001	0.000	0.000	0.027		0.000	0.000
	P3	1.000	0.999	1.000	1.000	0.866	0.000		1.000
	P4	1.000	1.000	1.000	1.000	0.953	0.000	1.000	

Scheffe test: variable Pollen Count (Pollen Count), Probabilities for Post Hoc Tests, Error: Between MS = 31,913, df = 199.00, n = 200. P1, P2, P3, and P4 indicate four randomly selected pollen subpopulations.

**Table 7:** Pollen count, forward scattered (FSC) and side scattered (SSC) of the commercial Taiwanese and Thai longan honeys.

Place of Origin	Pollen count (M ± SE) *	FSC (a.u) (M ± SE) *	SSC (a.u) (M ± SE) *
Artificial honey	00 ± 00 ^a^	00 ± 00 ^a^	00 ± 00 ^a^
Taiwan	3855.00 ± 204.24 ^b^	6.82 × 10^5^ ± 3.39 × 10^3 b^	1.66 × 10^6^ ± 1.57 × 10^4 b^
Thailand	339.00 ± 88.22 ^c^	6.48 × 10^5^ ± 1.87 × 10^4 c^	2.24 × 10^6^ ± 4.53 × 10^4 c^

FSC = forward scattered fluorescence measuring the size of pollen; SSC = side scattered fluorescence indicating pollen granularity. a.u = arbitrary units. * Means within a column followed by the same letter are not significantly different (α = 0.05) (Tukey HSD test). M = mean, SE = standard error, n = 15.

## Data Availability

The original contributions presented in the study are included in the article, further inquiries can be directed to the corresponding author.

## References

[1] Chaikham P., Prangthip P. (2015). Alteration of Antioxidative Properties of Longan Flower-Honey After High Pressure Ultra-sonic and Thermal Processing. Food Biosci..

[2] Chanchao C., Sintara K., Wongsiri S. (2006). Comparison of Antibiotic and Organoleptic Properties of Honey from Various Plant Sources in Thailand. J. Apic. Sci..

[3] Chen C., Chen B., Nai Y., Chang Y., Chen K., Chen Y. (2019). Novel Inspection of Sugar Residue and Origin in Honey based on the 13C/12C Isotopic Ratio and Protein Content. J. Food Drug Anal..

[4] Kafle L., Chinkangsadarn S. (2022). Beekeeping and bee product quality control in Taiwan. J. Entomol. Res..

[5] Lu M.C., Chantawannakul P., Williams G., Neumann P. (2018). Beekeeping on Taiwan Island. Asian Beekeeping in the 21st Century.

[6] Wang J., Li Q.X. (2011). Chemical Composition, Characterization, and Differentiation of Honey Botanical and Geographical Origins. Adv. Food Nutr. Res..

[7] Jamil Noor M., Ahmad M., Ashraf M.A., Zafar M., Sultana S. (2016). A Review of the Pollen Analysis of South Asian Honey to Identify the Bee Floras of the Region. Palynology.

[8] Heidmann I., Schade-Kampmann G., Lambalk J., Ottiger M., Di Berardino M. (2016). Impedance flow cytometry: A novel technique in pollen analysis. PLoS ONE.

[9] Gierlicka I., Kasprzyk I., Wnuk M. (2022). Imaging Flow Cytometry as a Quick and Effective Identification Technique of Pollen Grains from *Betulaceae*, *Oleaceae*, *Urticaceae* and *Asteraceae*. Cells.

[10] Cossarizza A., Chang H., Radbruch A., Akdis M., Andrä L., Annunziato F., Baumgart S. (2017). Guidelines for the Use of Flow Cytometry and Cell Sorting in Immunological Studies. Eur. J. Immunol..

[11] Pospichalova V., Svoboda J., Dave Z., Kotrbova A., Kaiser K., Klemova D., Minar L. (2015). Simplified Protocol for Flow Cytometry Analysis of Fluorescently Labeled Exosomes and Microvesicles Using Dedicated Flow Cytometer. J. Extracell. Vesicles.

[12] Kron P., Loureiro J., Castro S., Čertner M. (2021). Flow cytometric analysis of pollen and spores: An overview of applications and methodology. Cytom. Part A.

[13] Dunker S., Motivans E., Rakosy D., Boho D., Maeder P., Hornick T., Knight T.M. (2021). Pollen analysis using multispectral imaging flow cytometry and deep learning. New Phytol..

[14] Kadluczka D., Sliwinska E., Grzebelus E. (2022). Combining genome size and pollen morphology data to study species relationships in the genus *Daucus* (Apiaceae). BMC Plant Biol..

[15] Koutecký P., Smith T., Loureiro J., Kron P. (2023). Best practices for instrument settings and raw data analysis in plant flow cytometry. Cytom. Part A.

[16] Kanwal M., Gogoi N., Jones B., Bariana H., Bansal U., Ahmad N. (2022). Pollen: A potential explant for genetic transformation in wheat (*Triticum aestivum* L.). Agronomy.

[17] Steinhoff C., Pickarski N., Litt T., Hajdas I., Welte C., Wurst P., Kühne D., Dolf A., Germer M., Kallmeyer J. (2022). New Approach to Separate and Date Small Spores and Pollen from Lake Sediments in Semi-Arid Climates. Radiocarbon.

[18] Barnes C.M., Power A.L., Barber D.G., Tennant R.K., Jones R.T., Lee G.R., Hatton J., Elliott A., Zaragoza-Castells J., Haley S.M. (2023). Deductive automated pollen classification in environmental samples via exploratory deep learning and imaging flow cytometry. New Phytol..

[19] Kron P., Kwok A., Husband B.C. (2014). Flow cytometric analysis of pollen grains collected from individual bees provides information about pollen load composition and foraging behaviour. Ann. Bot..

[20] Baksay S., Pornon A., Burrus M., Mariette J., Andalo C., Escaravage N. (2020). Experimental quantification of pollen with DNA metabarcoding using ITS1 and trnL. Sci. Rep..

[21] Baydar H., Tuğlu Ü. (2024). Comparing nuclear DNA content, pollen viability, pollen production and seed retention of lavender and lavandin. Mediterr. Agric. Sci..

[22] Kron P., Husband B.C. (2012). Using flow cytometry to estimate pollen DNA content: Improved methodology and applications. Ann. Bot..

[23] Luria G., Rutley N., Lazar I., Harper J.F., Miller G. (2019). Direct analysis of pollen fitness by flow cytometry: Implications for pollen response to stress. Plant J..

[24] Lai L.-W. (2022). Influence of warming climate and the green revolution on the optimum range of weather parameters of longan yield in Taiwan since 1909. Theor. Appl. Climatol..

[25] Sopadang A., Tippayawong K.Y., Chaowarut W. (2012). Application of value chain management to longan industry. Am. J. Agric. Biol. Sci..

[26] Bicudo de Almeida-Muradian L., Barth O.M., Dietemann V., Eyer M., Freitas A.D., Martel A.C., Sattler J.A.G., Marcazzan G.L., Marchese C.M., Mucignat-Caretta C. (2020). Standard Methods for *Apis mellifera* Honey Research. J. Apic. Res..

[27] AWssociation of Official Analytical Chemists (2000). Official Methods of Analysis.

[28] Miwa N., Yokoyama K., Wakabayashi H., Nio N. (2010). Effect of Deamidation by Protein-Glutaminase on Physicochemical and Functional Properties of Skim Milk. Int. Dairy J..

[29] Aljohar H.I., Maher H.M., Albaqami J., Al-Mehaizie M., Orfali R., Orfali R., Alrubia S. (2018). Physical and chemical screening of honey samples available in the saudi market: An important aspect in the authentication process and quality assessment. Saudi Pharm. J..

[30] Durmishi B., Knights V., Mehmeti I., Stamatovska V., Nuha D., Rizani S., Bytyçi P., Haziri V., Sadiku V. (2023). Determining the quality of honey in the region of Kosova with physiochemical analysis. Uludağ Arıcılık Derg..

[31] Cavdar S., Yıldız O., Şahin H., Karahalil F., Kolaylı S. (2013). Comparison of physical and biochemical characteristics of different quality of Turkish honey. Uludağ Bee J..

[32] Narjes M.E., Lippert C. (2021). Regional differences in farmers’ preferences for a native bee conservation policy: The case of farming communities in Northern and Eastern Thailand. PLoS ONE.

[33] Royo V.D.A., Oliveira D.A.d., Veloso P.H.F., Sacramento V.D.M., Olimpio E.L., Souza L.F.d., Pires N.D.C., Martins C.H.G., Santiago M.B., Alves T.M.D.A. (2022). Physicochemical profile, antioxidant and antimicrobial activities of honeys produced in Minas Gerais (Brazil). Antibiotics.

[34] Sant’Ana L.D., Ferreira A.B.B., Lorenzon M.C., Berbara R.L., Castro R.N. (2014). Correlation of Total Phenolic and Flavonoid Contents of Brazilian Honeys with Colour and Antioxidant Capacity. Int. J. Food Prop..

[35] Guede S.S., Yeo D.M., Soro Y.R., Toure A. (2022). Physicochemical characterization of local honeys marketed in Korhogo tow. GSC Biol. Pharm. Sci..

[36] Manickavasagam G., Saaid M., Lim V. (2024). Impact of prolonged storage on quality assessment properties and constituents of honey: A systematic review. J. Food Sci..

[37] Dar S.A., Farook U.B., Rasool K., Ahad S. (2024). Honey: Classification, composition, safety, quality issues and health benefits. Advanced Techniques of Honey Analysis.

[38] Bogdanov S., Martin P., Lullmann C. (1997). Harmonized Methods of the International Honey Commission. Apidologie (extra issue).

[39] Louveaux J., Maurizio A., Vorwohl G. (1978). Methods of Melissopalynology. Bee World.

[40] Reardon A.J., Elliott J.A., McGann L.E. (2014). Fluorescence as an alternative to light-scatter gating strategies to identify frozen–thawed cells with flow cytometry. Cryobiology.

[41] Mouhoubi-Tafinine Z., Ouchemoukh S., Louaileche H., Tamendjari A. (2018). Effect of storage on hydroxymethylfurfural (HMF) and color of some Algerian honey. Int. Food Res. J..

[42] Rao Z., Hua D., He T., Wang Q., Le J. (2017). Ultraviolet laser-induced fluorescence lidar for pollen detection. Optik.

[43] Fukuda N. (2023). Apparent diameter and cell density of yeast strains with different ploidy. Sci. Rep..

[44] Jo J., Hugonnet H., Lee M.J., Park Y. (2024). Digital cytometry: Extraction of forward and side scattering signals from holotomography. arXiv.

[45] Ortolani C. (2022). Signals: Scattering. Flow Cytometry Today: Everything You Need to Know about Flow Cytometry.

[46] Sandmann M., Rading M. (2024). Starch granules in algal cells play an inherent role to shape the popular SSC signal in flow cytometry. BMC Res. Notes.

[47] Pauliuc D., Ciursă P., Ropciuc S., Dranca F., Oroian M. (2021). Physicochemical parameters prediction and authentication of different monofloral honeys based on FTIR spectra. J. Food Compos. Anal..

[48] Yildiz O., Gurkan H., Sahingil D., Degirmenci A., Er Kemal M., Kolayli S., Hayaloglu A.A. (2022). Floral authentication of some monofloral honeys based on volatile composition and physicochemical parameters. Eur. Food Res. Technol..

[49] (2016). Chinese National Standards for Honey.

[50] Gün R., Karaoğlu M.M. (2024). Detection of honey adulteration by characterization of the physico-chemical properties of honey adulterated with the addition of glucose–fructose and maltose corn syrups. Eur. Food Res. Technol..

[51] Çobanoğlu D.N., Akyıldız İ.E., Kızılpınar Temizer İ., Damarlı E., Çelik Ş. (2023). Phenolic compound, organic acid, mineral, and carbohydrate profiles of pine and blossom honeys. Eur. Food Res. Technol..

[52] Seraglio S.K.T., Silva B., Bergamo G., Brugnerotto P., Gonzaga L.V., Fett R., Costa A.C.O. (2019). An overview of physicochemical characteristics and health-promoting properties of honeydew honey. Food Res. Int..

[53] Cheung Y., Meenu M., Yu X., Xu B. (2019). Phenolic acids and flavonoids profiles of commercial honey from different floral sources and geographic sources. Int. J. Food Prop..

[54] Mongi R.J. (2024). Influence of botanical origin and geographical zones on physicochemical properties, mineral contents and consumer acceptance of honey in Tanzania. Food Chem. Adv..

[55] Bogdanov S., Ruoff K., Oddo L.P. (2004). Physico-Chemical Methods for the Characterisation of Unifloral Honeys: A Review. Apidologie.

[56] Srisayam M., Chantawannakul P. (2010). Antimicrobial and Antioxidant Properties of Honeys Produced by *Apis mellifera* in Thailand. J. ApiProd. ApiMed. Sci..

[57] Sakdatorn V. (2017). The Effects of Magnetic Fields on Viscosity, Color and pH of Longan Honey. Naresuan Univ. Eng. J..

[58] González-Miret M.L., Terrab A., Hernanz D., Fernández-Recamales M.A., Heredia F.J. (2005). Multivariate Correlation between Color and Mineral Composition of Honeys and by Their Botanical Origin. J. Agric. Food Chem..

[59] Bodor Z., Benedek C., Urbin Á., Szabó D., Sipos L. (2021). Colour of honey: Can we trust the Pfund scale?—An alternative graphical tool covering the whole visible spectra. LWT.

[60] Moniruzzaman M., Sulaiman S.A., Khalil M.I., Gan S.H. (2013). Evaluation of Physicochemical and Antioxidant Properties of Sourwood and Other Malaysian Honeys: A Comparison with Manuka Honey. Chem. Cent. J..

[61] Mossel B., Bhandari B., D’Arcy B., Caffin N. (2003). Determination of Viscosity of Some Australian Honeys Based on Composition. Int. J. Food Prop..

[62] Gómez-Díaz D., Navaza J.M., Quintáns-Riveiro L.C. (2009). Effect of Temperature on the Viscosity of Honey. Int. J. Food Prop..

[63] Conforti P.A., Lupano C.E., Malacalza N.H., Arias V., Castells C.B. (2006). Crystallization of Honey at −20 °C. Int. J. Food Prop..

[64] Bogdanov S., Martin P. (2002). Honey authenticity: A review. Mitt. Lebensm. Hyg..

[65] Neupane B.P., Gautam A., Malla K.P. (2020). Non-acetolysed pollen analysis of mad honey from the Himalayas, Nepal. Bee World.

[66] Silici S., Gökceoglu M. (2007). Pollen analysis of honeys from Mediterranean region of Anatolia. Grana.

[67] Chaimanee V., Chantawannakul P., Khongphinitbunjong K., Kamyo T., Pettis J.S. (2019). Comparative Pesticide Exposure to *Apis mellifera* via Honey Bee-collected Collen in Agricultural and Non-Agricultural Areas of Northern Thailand. J. Apic. Res..

[68] Chantawannakul P., Chantawannakul P., Williams G., Neumann P. (2018). Bee Diversity and Current Status of Beekeeping in Thailand. Asian Beekeeping in the 21st Century.

[69] Wilczyńska A. (2014). Effect of filtration on colour, antioxidant activity and total phenolics of honey. LWT Food Sci. Technol..

[70] Wagley S., Morcrette H., Kovacs-Simon A., Yang Z.R., Power A., Tennant R.K., Love J., Murray N., Titball R.W., Butler C.S. (2021). Bacterial dormancy: A subpopulation of viable but non-culturable cells demonstrates better fitness for revival. PLoS Pathog..

[71] Pöhlker C., Huffman J.A., Förster J.-D., Pöschl U. (2013). Autofluorescence of atmospheric bioaerosols: Spectral fingerprints and taxonomic trends of pollen. Atmos. Meas. Tech..

[72] Bryant V. (2017). Filtering honey almost every filter removes some pollen. Bee Cult. Mag. Am. Beekeep.

[73] Moon H.S., Eda S., Saxton A.M., Ow D.W., Stewart C.N. (2011). An efficient and rapid transgenic pollen screening and detection method using flow cytometry. Biotechnol. J..

